# The Choice of a Health Resort

**Published:** 1899-05-27

**Authors:** 


### THE CHOICE OF A HEALTH RESORT.

At the last meeting of the British Balneological and Climatological Society, Sir Hermann Weber, in drawing a comparison between the climatic health resorts of the United Kingdom and those on the Continent, said that in regard to marine resorts there was no country which could compete with Great Britain during the summer and autumn, and that even during winter several English sea-coast places offered—at all events to some constitutions—advantages over Continental resorts. Our inland climates, however, are more limited in character than our sea climates, and the Continent has an advantage over Great Britain with regard to the different degrees of elevation, of humidity, of isolation, and of shelter which can be met with. It has to be borne in mind, however, that although England may not always offer exactly what is wanted for the treatment of an invalid it is by no means therefore to be inferred, as some would have it, that the English climate is a bad one. The climates of England are, it is true, not the most exhilarating and agreeable, but the brightest climates, like those of Egypt, Spain, Italy, and Greece, are certainly not the best for health and strength.

"A climate with constant moderate variations in its principal factors is the best for the maintenance of health. It calls forth the energy of the different organs and functions, their power of adaptation and resistance, and keeps them in working order." Such, says Sir Hermann Weber, are the climates of England, and they belong to the most health-giving in the world. They produce the finest animals, the finest trees, and the finest men and women, and are most conducive to health and longevity. In order, however, that invalids may get all the benefits which can be gained from our climate, it is necessary that the authorities at our health resorts should take care to provide glass and other shelters, so that visitors may be protected from the cold wind, and may receive in full such sunshine as is to be obtained. "It is not only in tuberculosis," he says, "that 'open-air treatment' is very useful, but in almost every chronic complaint. I have for many years recommended it with due precautions in anæmia, in neurasthenia, general debility, tendency to rheumatic and catarrhal affections, and so on."

The choice of a health resort, however, can but seldom depend entirely upon the mere question of what is the best climate for the case. Many other matters require to be taken into consideration. "We must always bear in mind not only the purely climatic qualities of the place, but also the nature of the accommodation there, including the cookery, the class of people frequenting it, the social conditions and amusements, and the manner in which the sum total of agencies, climatic and social, is likely to influence the person who asks our advice. . . . We may even have to sacrifice some climatic advantages for certain invalids if we have reason to suppose that they will become depressed and unhappy at a certain climatically suitable place. Experience abundantly shows us that, however good the climatic elements may be, mental dulness causes in certain types of people manifold functional troubles, such as want of appetite, dyspepsia, imperfect action of the bowels, weakness and irregularity of the heart's action, inability to take exercise, sleeplessnesss, and loss of weight." Clearly, the choice of a place for an invalid is not a matter of merely summing up the weather reports. Still, mere climate has much to do even with happiness; for there are people who, although they are happy and well so long as they are in a warm and sunny locality, yet become depressed and lose their appetite, become sleepless and cannot work, have urine loaded with lithates and lose weight if they are obliged to be at a place which is cold, damp, misty, sunless, and without music, flowers, or butterflies. "There are many persons, principally so-called 'people without nerves,' who cannot understand and appreciate such differences in the condition of delicate persons. These conditions, however, are not imaginary; they are fearfully real, and ought not to be overlooked in giving advice about climatic resorts."

